# Uncovering the Microbiota of Bagworm *Metisa plana* (Lepidoptera: Psychidae) in Oil Palm Plantations in Malaysia

**DOI:** 10.21315/tlsr2023.34.1.11

**Published:** 2023-03-31

**Authors:** Andrew Ting, Cik Mohd Rizuan Zainal Abidin, Noor Hisham Hamid, Ghows Azzam, Hasber Salim

**Affiliations:** 1 School of Biological Sciences, Universiti Sains Malaysia, 11800 USM, Pulau Pinang, Malaysia; 2 Pest Management Unit, FGV R&D Sdn Bhd, Pusat Penyelidikan Pertanian Tun Razak, 26400 Bandar Jengka, Pahang, Malaysia

**Keywords:** Metagenomics, Microbiome, *Metisa plana*, Bagworm, Oil Palm, Metagenomik, Mikrobiom, *Metisa plana*, *Bagworm*, Kelapa Sawit

## Abstract

Bagworm *Metisa plana* is one of the major pests in Malaysia’s oil palm plantation, with infestation resulting in huge economical loss. Currently, the microbial profile of the bagworm has yet to be study. Understanding the biology of the pest such as the bacterial community is crucial as bacteria associated with insects often provide benefits to the insect, giving the insect host a better chance of survival. Here, 16S amplicon sequencing was used to identify the bacteria community of *M. plana.* Additionally, two comparisons were made, the bacterial communities between two larval stages (early instar stage and late instar stage) from outbreak area; the bacterial communities of late instar stage larvae from non-outbreak between outbreak areas. From this study, it was found that the bacterial community of *M. plana* consisted of *Proteobacteria*, *Actinobacteria*, *Bacterioidetes*, *Firmicutes* and other minor phyla, with *Proteobacteria* being the most dominant phylum. Furthermore, bacterial genera of *M. plana* consisted of *Pantoea*, *Curtobacterium*, *Pseudomonas*, *Massilia* and other minor genera, with *Pantoea* being the most dominant. It was also found that the alpha and beta diversity in both comparisons were not significantly different. We present our data as a first insight towards the bacterial community of *M. plana*, paving a way towards understanding the biology of the bagworm *M. plana*.

## Abstract (fr)

*Metisa plana* adalah salah satu daripada perosak utama dalam ladang kelapa sawit Malaysia, dengan serangan yang mengakibatkan kerugian besar kepada ekonomi. Pada masa ini, profil mikrob *bagworm* masih belum dikaji. Memahami biologi perosak seperti komuniti bakteria adalah penting kerana bakteria yang dikaitkan dengan serangga sering memberi manfaat kepada serangga, memberikan hos serangga peluang untuk terus hidup. Penjujukan amplikon 16S digunakan untuk mengenal pasti komuniti bakteria *M. plana*. Selain itu, dua perbandingan telah dibuat, komuniti bakteria antara dua peringkat larva (peringkat instar awal dan peringkat instar lewat) dari kawasan wabak; komuniti bakteria larva peringkat instar lewat dari kawasan bukan wabak dan kawasan wabak. Daripada kajian ini, didapati komuniti bakteria *M. plana* terdiri daripada *Proteobacteria*, *Actinobacteria, Bacterioidetes, Firmicutes* dan filum kecil lain, dengan *Proteobacteria* merupakan filum yang paling dominan. Tambahan pula, genera bakteria *M. plana* terdiri daripada *Pantoea, Curtobacterium, Pseudomonas, Massilia* dan genera minor lain, dengan *Pantoea* yang paling dominan. Kajian ini juga mendapati bahawa kepelbagaian alfa dan beta dalam kedua-dua perbandingan adalah tidak jauh berbeza. Data ini dibentangkan sebagai pandangan pertama terhadap komuniti bakteria *M. plana*, membuka jalan ke arah memahami biologi *bagworm M. plana*.

**Highlights**
- Bacterial community profile of *Metisa plana* from different developmental stages and different areas were identified and compared.
- Bacterial community of *M. plana* was dominated by *Proteobacteria* phylum and *Pantoea* genus.
- No significant difference in bacterial community of *M. plana* between developmental stages as well as between areas.

## INTRODUCTION

The Lepidoptera is a vastly diverse insect order, with many species considered as major pests of agricultural importance (González-Serrano *et al*. 2020). The Lepidopteran pest bagworm is the most serious and economically important pests in the oil palm plantations in Malaysia (Cheong & Tey 2012; Kamarudin & Wahid 2007; Kok *et al*. 2011; Salim *et al*. 2015; Wood 1968). The bagworm outbreak can result in a terrible yield loss which can translate into millions of Ringgit Malaysia (Malaysia’s local currency) (Ahmad Ali *et al*. 2011; Salim *et al*. 2015). Of the common species of bagworm found in the oil plantations (*Mahasena corbetti*, *Pteroma pendula* and *Metisa plana*), the *M. plana* is the most serious leaf defoliator (Ahmad Ali *et al*. 2011; Sankaran 1970; Wood 1968). Although there are available and effective control measures (Salim *et al*. 2015; Salim & Hamid 2012; Wood 2019; Yap 2000) the outbreak and infestation of the bagworm is still an occurring problem due to the lack of understanding of the pests (Cheong & Tey 2012; Kok *et al*. 2011).

Huge ranges of microorganisms colonise the insects, from the largest of fungi to the smallest of virus. The microbiota composition of the insects differs greatly and are affected by different factors such as insect developmental stages, environments, and even diet (Chaturvedi *et al*. 2017; Hammer *et al.* 2014; Mereghetti *et al.* 2017; Voirol *et al*. 2018). Often times, these microorganisms provide various benefits to the wellbeing of the insect, but sometimes may be pathogenic (De Smet *et al*. 2018; Douglas 2015; Morimoto *et al*. 2019; Voirol *et al*. 2018). An example of benefits from insect-bacteria interaction is the acquisition of nutrients. Chewing insects that feed on leaves would not have enough nitrogen solely from their diet. This insufficient nitrogen obtained from the diet would be supplemented by bacterial symbionts which can fix nitrogen and convert it into appropriate nitrogen-containing compounds (Hansen *et al.* 2020; Nardi *et al.* 2002; Voirol *et al*. 2018). Some symbiotic bacteria could also protect the host against pathogens. In a separate study, Shao showed that the dominant symbiotic bacterium *Enterococcus mundtii* actively secretes bacteriocin against bacterial invaders. This interaction protects the host from other invading bacteria and at the same time, provides the bacterium an advantage which contributed to its dominance (Shao *et al*. 2017).

The bacterial community of the *M. plana* bagworm to the best of the authors’ knowledge has yet to be explored. The current study therefore aims at identifying and compare the bacterial community of the insect host. This knowledge can help to further understand the biology of the pest, and could potentially be used to improve on the integrated pest management methods such as using microbes as a biocontrol agent (Charles *et al.* 1996; Federici 2005; 2007; Köhl *et al.* 2019). Here, we used 16S rRNA amplicon sequencing to investigate the bacterial community of *M. plana* and to see whether there is any difference in the bacterial community: (1) between the early instar stage and late instar stage *M. plana* larvae from the outbreak area; and (2) between the late instar stage *M. plana* larvae from non-outbreak area and outbreak area.

## MATERIALS AND METHODS

### Samplings

The *M. plana* larvae of late instar stage was collected in the month of August 2020, from non-outbreak area located in Felda Jengka 7, Jengka, Pahang, Malaysia. *M. plana* larvae of both early instar stage (1st instar to 3rd instar) and late instar stage (4th instar to 6th instar) were collected in the month of September 2019 from outbreak area located in Felda Gunung Besout 02/03, Trolak, Perak, Malaysia. The instar stage of *M. plana* larvae was determined by the length and morphology of the case as described by Kok *et al*. (2011). The outbreak area is categorized by the persistent infestation of bagworm larvae of more than the economic threshold level (ETL), which is five larvae per frond (Salim *et al*. 2015).

### Ethics Statements

This species is a pest and is not protected by law. Bagworm was declared a dangerous pest under the Malaysia Act 167, Plant Quarantine Act 1976 (Kamarudin *et al*. 2017). Sampling was performed with proper protective equipment to ensure no contamination from and to the bagworm samples.

### Total DNA Extraction

Genomic DNA (gDNA) was extracted using Qiagen DNeasy Blood and Tissue Kit (Cat No./ID: 69506) with slight modifications in 4 replicates for each group (late instar stage larvae from non-outbreak area, early instar stage and late instar stage larvae from outbreak area). For each replicate, 20 whole bagworms were removed from their bags and surface sterilised before being placed in 1.5 mL microcentrifuge tube before adding 180 μL of ATL buffer. The samples were then kept at –20°C for 30 min before being homogenised using micropipette tips. Twenty microlitre (20 μL) of proteinase K was added to the sample and mixed by vortexing before the samples were incubated at 56°C for 10 min. The samples were then vortexed for 15 sec before adding 200 μL of AL buffer. The samples were mixed by vortexing and incubated at 56°C for 10 min. Ice-cold absolute ethanol of 200 μL was added to the samples and mixed. The samples were centrifuged at 6,000 × g for 1 min and the supernatant were transferred to DNeasy Mini spin column. The spin columns were then centrifuged at 6,000 × g for 1 min. The spin columns were placed in a new 2 mL collection tubes and 500 μL of Buffer AW1 was added before centrifuging for 1 min at 6,000 × g. The spin columns were again placed in new 2 mL collection tubes and added with 500 μL of Buffer AW2 before centrifuging at 13, 200 × g for 8 min. The spin columns were placed in new 1.5 mL microcentrifuge tubes and 50 μL of Buffer AE was added directly to the spin columns’ membranes. They were then incubated for 3 min at room temperature before centrifuging at 6,000 × g for 1 min. The eluates were pipetted back into the spin column’s membrane and incubated for 3 min before centrifuging at 6,000 × g for 1 min. Gel electrophoresis was performed and the results were visualised under ultraviolet light.

### Library Preparation and 16S Amplicon Sequencing

The extracted gDNA were sent to the sequencing service provider, Apical Scientific Sdn Bhd (https://apicalscientific.com/) for library preparation and sequencing. V3–V4 variable regions of the 16S ribosomal RNA gene was amplified using the forward primer (5′ CCTACGGGNGGCWGCAG) and reverse primer (5′ GACTACHVGGGTATCTAATCC). After passing the quality check, the V3–V4 variable region were amplified using locus-specific sequence primers with overhang adapters (forward overhang 5′ TCGTCGGCAGCGTCAGATGTGTATAAGAGACAG- [locus-specific sequence]; reverse overhang 5′ GTCTCGTGGGCTCGGAGATGTGTATAAGAGACAG-[locus-specific sequence]). All the PCR reactions were carried out with Q5® Hot Start High-Fidelity 2X Master Mix.

### Analysis of Microbial Community

#### Sequence analysis

The analysis was done using Mothur software (v.1.44.3) (Schloss *et al*. 2009) with adaptations from MiSeq standard operating procedure (SOP) (https://mothur.org/wiki/miseq_sop/) (Kozich *et al*. 2013). The forward reads and reverse reads were merged, and primers were removed. Sequences that were longer than 440 base pair (bp), but shorter than 406 bp, and with any ambiguities were removed. Duplicates sequences and sequences that only appeared once were also removed. A customized reference targeting the V3–V4 region of the 16S rRNA gene was made from SILVA Seed v132 (Quast *et al*. 2013). Unique sequences were then aligned to the customised refence. Sequences that start before position 2 and ends after 17012, with homopolymer more than 8 as well as a length shorter than 406 bp were removed before removing gap characters. The sequences were pre-clustered, and chimeras were removed. The remaining sequences were classified to SILVA reference database using Bayesian classifier at 80% confidence threshold. Sequences that were classified into “Chloroplast”, “Mitochondria”, “Unknown”, “Archaea” and “Eukaryote” were removed. The sequences with similarity of 97% were then clustered into operational taxonomical units (OTU).

### Bacterial community analysis

As the samples showed unequal sampling depth, we investigated the alpha and beta diversity of the bacterial communities using rarefied OTU tables. To access the alpha-diversity, we calculated the Shannon diversity index, observed species richness (Sobs) and Shannon evenness index. Wilcoxon test was performed using to see whether the alpha diversity as well as beta-diversity were significantly different. Principle Coordinate Analysis (PCoA) was plotted to visualise the cluster separation of the bacterial community’s structure. Analysis of Molecular Variance (AMOVA) was performed to see whether the centre of the cluster representing each group were significantly different. We performed Homogeneity of Molecular Variance (HOMOVA) to see whether the variation in each group were significantly different from each other. All statistical tests were performed with significance at adjusted *p*-value at 0.05.

## RESULTS

### Overview of the Bacterial Community in *M. plana* larvae

From the results of the study, it was observed that the bacterial community of *M. plana* was dominated by *Proteobacteria*, followed by *Actinobacteria, Bacterioidetes*, *Firmicutes* and other phyla which constitute a minor percentage of the bacterial community (Appendix A). At the bacterial family level, the most dominant family was the *Enterobacteriaceae*, followed by *Microbacteriaceae*, *Burkholderiaceae*, *Pseudomonadaceae*, *Sphingobacteriaceae* and other bacterial families, constituting a minor percentage in the bacterial community (Appendix B). At the genera level, the *Pantoea* genus was the dominant genus, followed by unclassified genus in the *Enterobacteriaceae* family, *Curtobacterium*, *Pseudomonas*, *Massilia*, and other minor genera (Appendix C).

### Comparison Between Early Instar and Late Instar Stage

**Figure 1:**
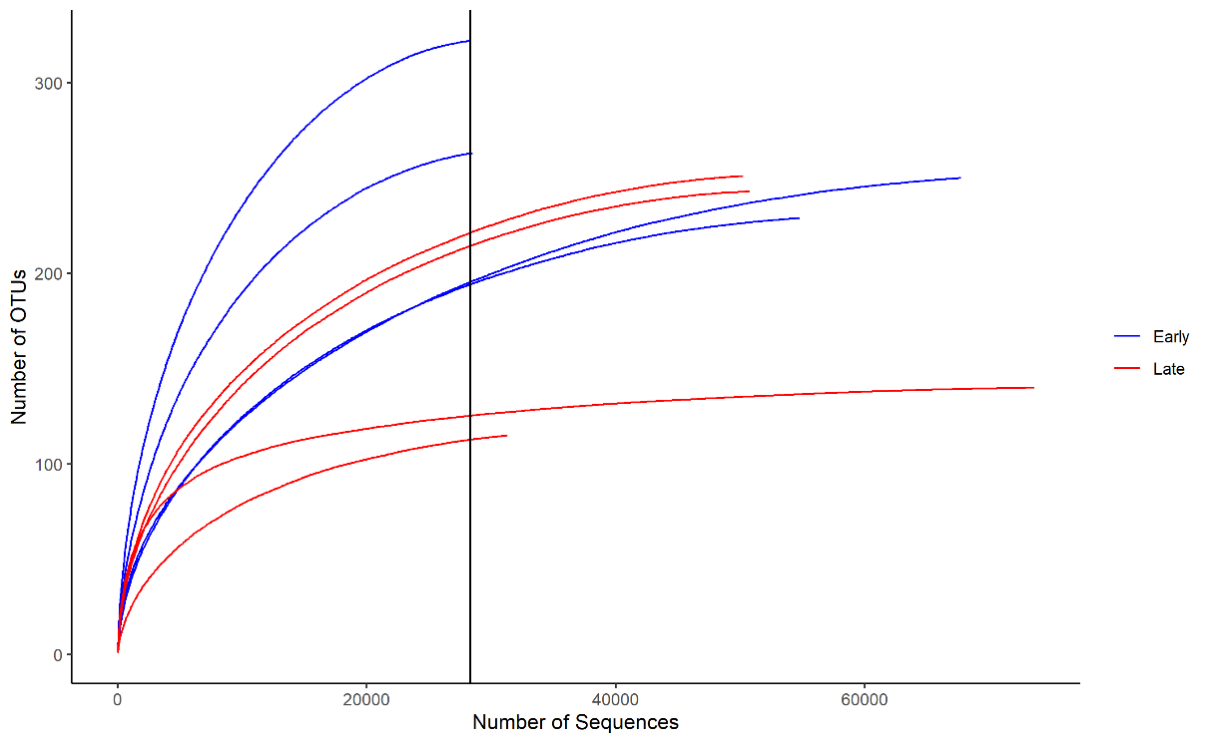
Rarefaction curve for the early instar stage and late instar stage samples. (x-axis intercept: samples were subsampled to 28,340 sequences).

To obtain the bacterial community composition of the *M. plana* larvae at early instar and late instar stage, the V3 and V4 region of the bacterial 16S rRNA gene was amplified. A total of 2,738,727 sequences were obtained from 8 samples. After quality checks and removing unwanted sequences, a total of 385,297 sequences with 3,757 unique sequences were obtained. The sequences were then clustered at 97% similarity into 959 Operational Taxonomical Units (OTUs). The rarefaction curve did not completely plateau (Fig. 1), suggesting the sequencing depth was insufficient to capture the entire bacterial community.

**Figure 2:**
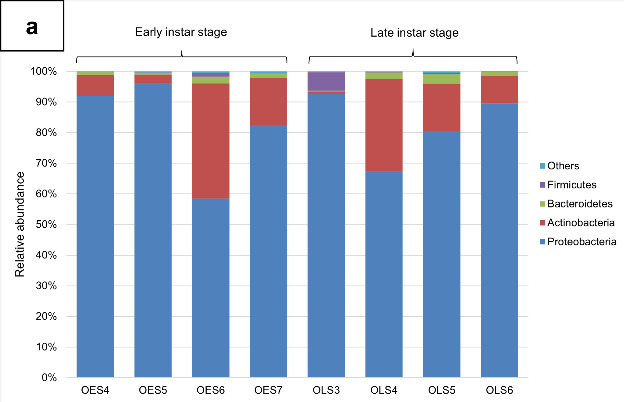

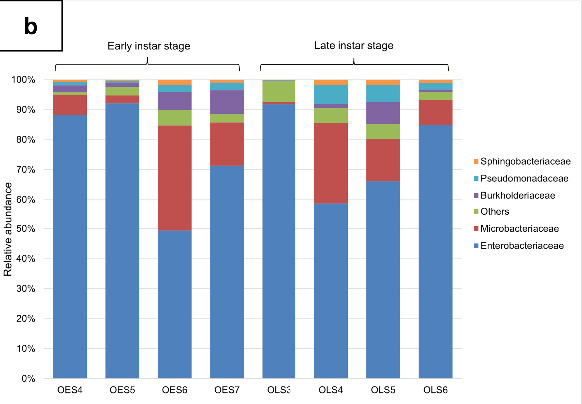

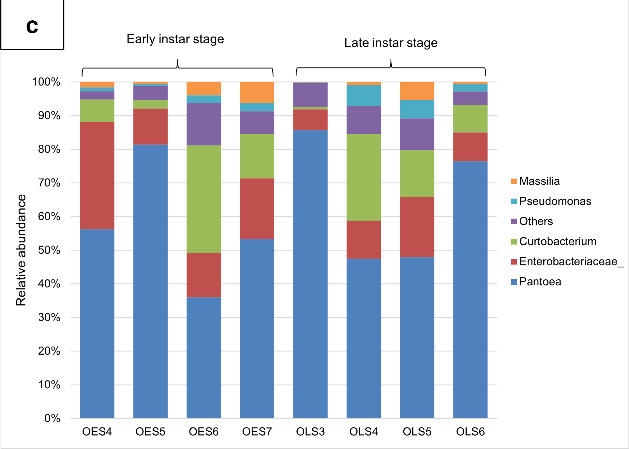
Bacterial community of early instar stage and late instar stage of *M. plana* larvae from outbreak area. (a) Bacterial phyla with average relative of more than 1%; (b) Bacterial families with with average relative of more than 1%; (c) Bacterial genera with average relative of more than 1%.

The bulk of the bacteria were of *Proteobacteria* (82.36%), *Actinobacteria* (14.8%), *Bacteroidetes* (1.48%), *Firmicutes* (1.01%) and remaining individual phyla consisting of less than 1% (Fig. 2a and Appendix A). Wilcoxon test showed no significant difference in relative abundance in any of the bacterial phyla between the two development stages. At family level, the *Enterobacteriaceae* was the dominant family (75.37%), followed by *Microbacteriaceae* (13.63%), *Burkholderiaceae* (3.4%), *Pseudomonadaceae* (2.56%), *Sphingobacteriaceae* (1.09%) and the remaining families individually having less than 1% relative abundance (Fig. 2b and Appendix B). Result showed no significant difference in relative abundance between the bacterial families (Appendix B). At genera level, the bacterial community was dominated by *Pantoea* with 60.57% average relative abundance, followed by unclassified *Enterobacteriaceae*, *Curtobacterium*, *Pseudomonas*, *Massilia* and remaining genera individually having less than 1% relative abundance (Fig. 2c and Appendix C). After performing Wilcoxon test, there were no significantly different bacterial genera (Appendix C).

Shannon diversity index, observed species richness and Shannon evenness were calculated to estimate the diversity of the bacterial community, the number of species and the evenness of the bacterial community (Table 1). However, result showed that the Shannon diversity index, sobs and evenness between the early instar stage and late instar stage were all not significantly different.

**Figure 3:**
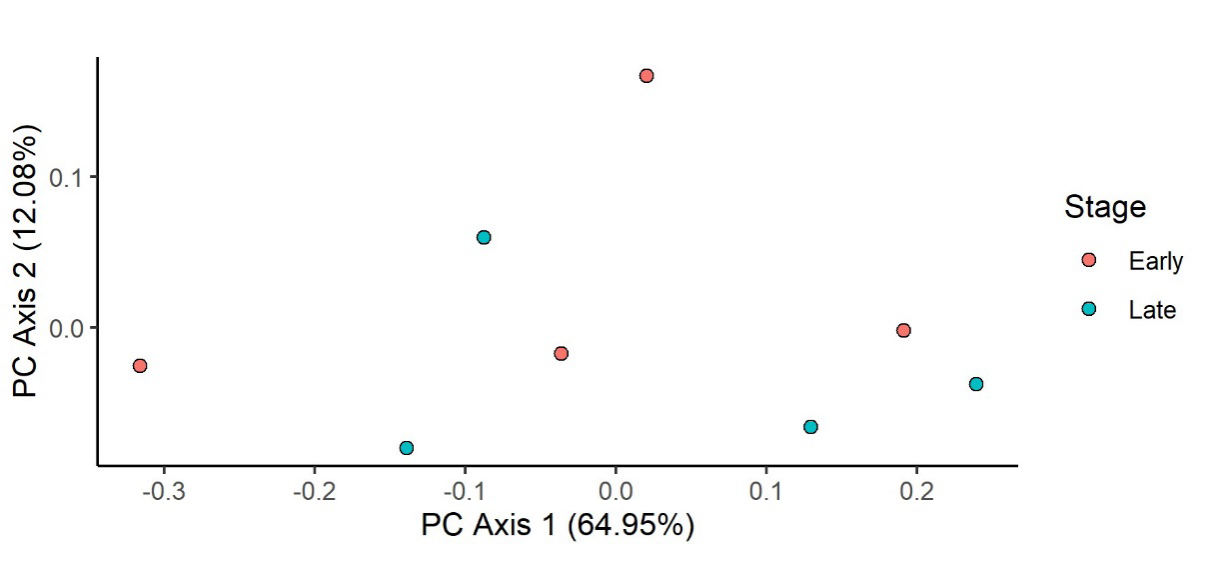
Principal Coordinate Analysis (PCoA) plot of bacterial communities of *M. plana* bagworm larvae in the comparison between early instar stage and late instar stage.

The PCoA was ordinated to visualise the cluster separation of the bacterial community. However, the ordination (Fig. 3) did not show clear separation between the early instar stage and late instar stage. AMOVA test was done on the samples to test whether the cluster of the early instar and late instar stage was significantly different. The result (Table 2) revealed that the observed separation in the early instar and late instar stage was not significantly different.

We also wanted to know whether the variation of the bacterial community in the early instar stage larvae was significantly different from that of the late instar stage. This was done by performing HOMOVA with the result (Table 3) showing no significant difference in the variation with the early instar stage and late instar stage.

### Comparison Between Non-Outbreak Area and Outbreak Area

**Figure 4:**
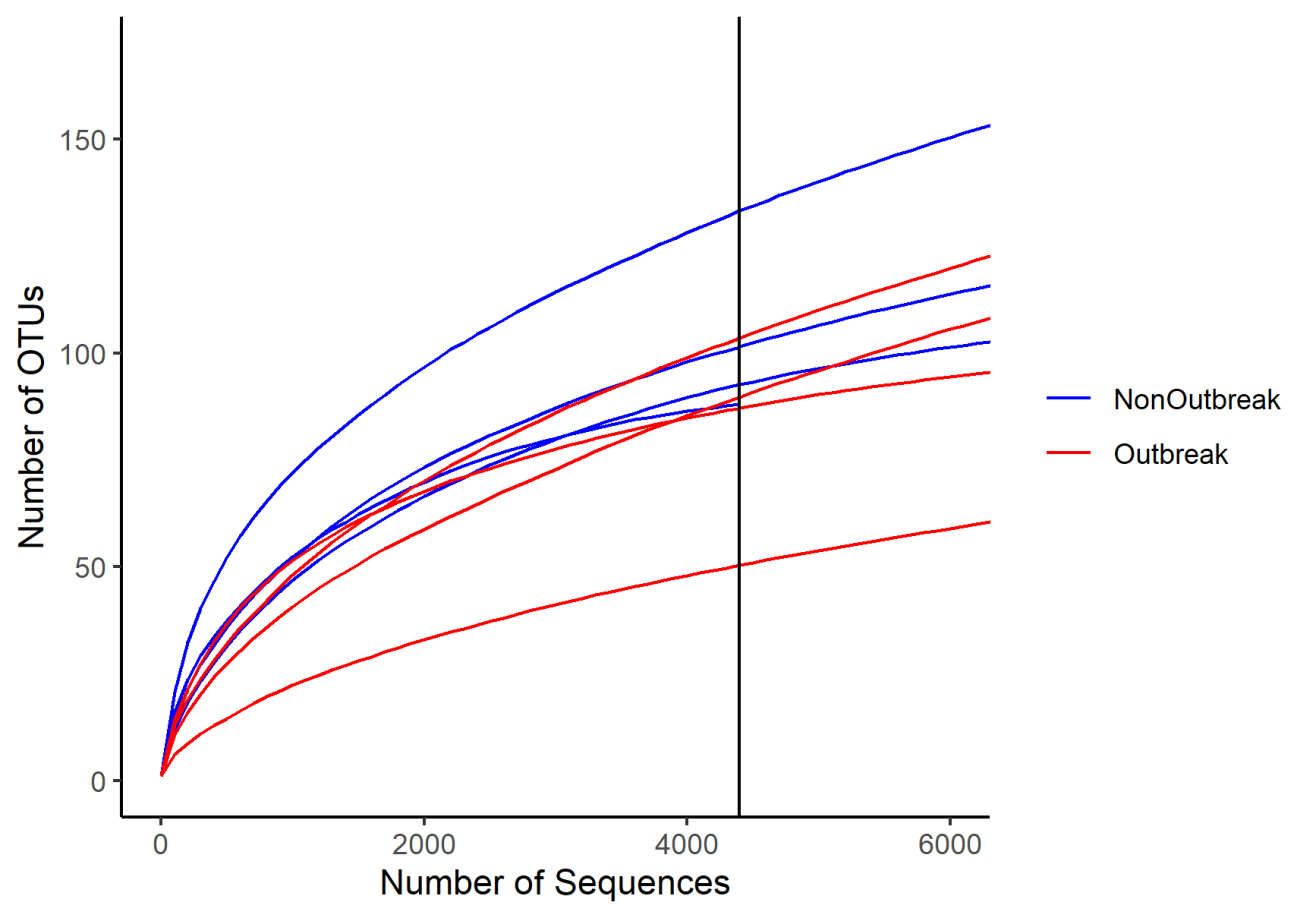
Rarefaction curve for the late instar stage samples from non-outbreak area and outbreak area. (x- axis intercept: samples were subsampled to 4,399 sequences). The curves showed the same number of sequences, the larvae from non-outbreak area had a greater number of OTUs than that of outbreak area.

The V3 and V4 region of the bacterial 16S rRNA gene was amplified using late instar stage larvae from the non-outbreak area and outbreak area. A total of 2,848,936 sequences were obtained from eight samples. After quality checks and removing unwanted sequences, a total of 271,821 sequences with 2,471 unique sequences were obtained. The sequences were then clustered at 97% similarity into 796 Operational Taxonomical Units (OTUs). The rarefaction curve did not plateau (Fig. 4), suggesting the sequencing depth was insufficient to capture the entire bacterial community.

**Figure 5:**
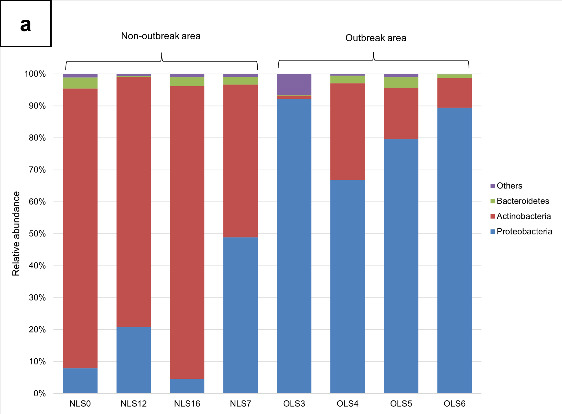

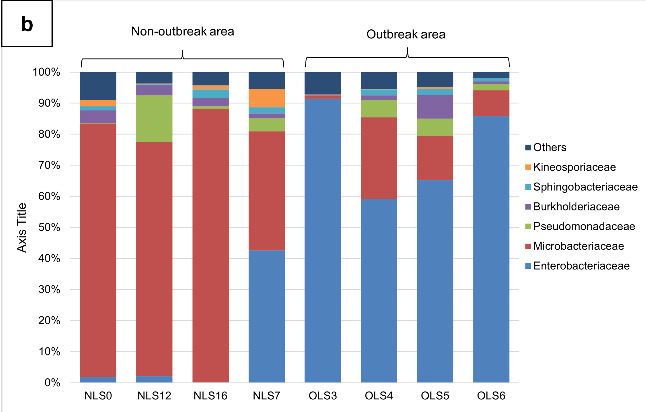

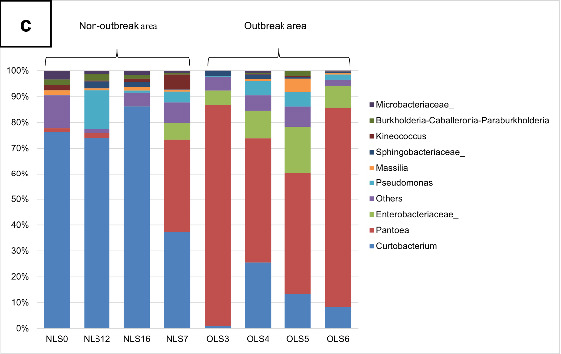
Bacterial community of the late instar stage of *M. plana* larvae from non-outbreak area and outbreak area. (a) Bacterial phyla with average relative of more than 1%; (b) Bacterial families with with average relative of more than 1%; (c) Bacterial genera with average relative of more than 1%.

The most abundant phyla consisted of *Proteobacteria* (51.30%) followed by *Actinobacteria* (45.22%), *Bacteroidetes* (1.98%) and the rest of the phyla individually consisting of less than 1% in relative abundance (Fig. 5a and Appendix D). After performing Wilcoxon test, we observed no significantly different bacterial phyla (Appendix D).

The most abundant families consisted of *Enterobacteriaceae* (43.54%), followed by *Microbacteriaceae* (41.67%), *Pseudomonadaceae* (4.18%), *Burkholderiaceae* (2.4%), *Sphingobacteriaceae* (1.74%), *Kineosporiaceae* (1.24%) and other families individually having less than 1% relative abundance pooled as “Others” (Fig. 5b and Appendix E). We again compared the relative abundance of families between the two areas and found that there were no significantly difference bacterial families (Appendix E). The most dominant bacterial genera that can be found were the *Curtobacterium* (40.24%) and *Pantoea* (37.29%) (Fig. 5c and Appendix F) but statistical test showed no significantly different bacterial genera. Shannon diversity index, observed species richness and Shannon evenness were calculated but result showed that the Shannon diversity index, sobs and evenness between the early instar stage and late instar stage were all not significantly different (Table 4).

**Figure 6:**
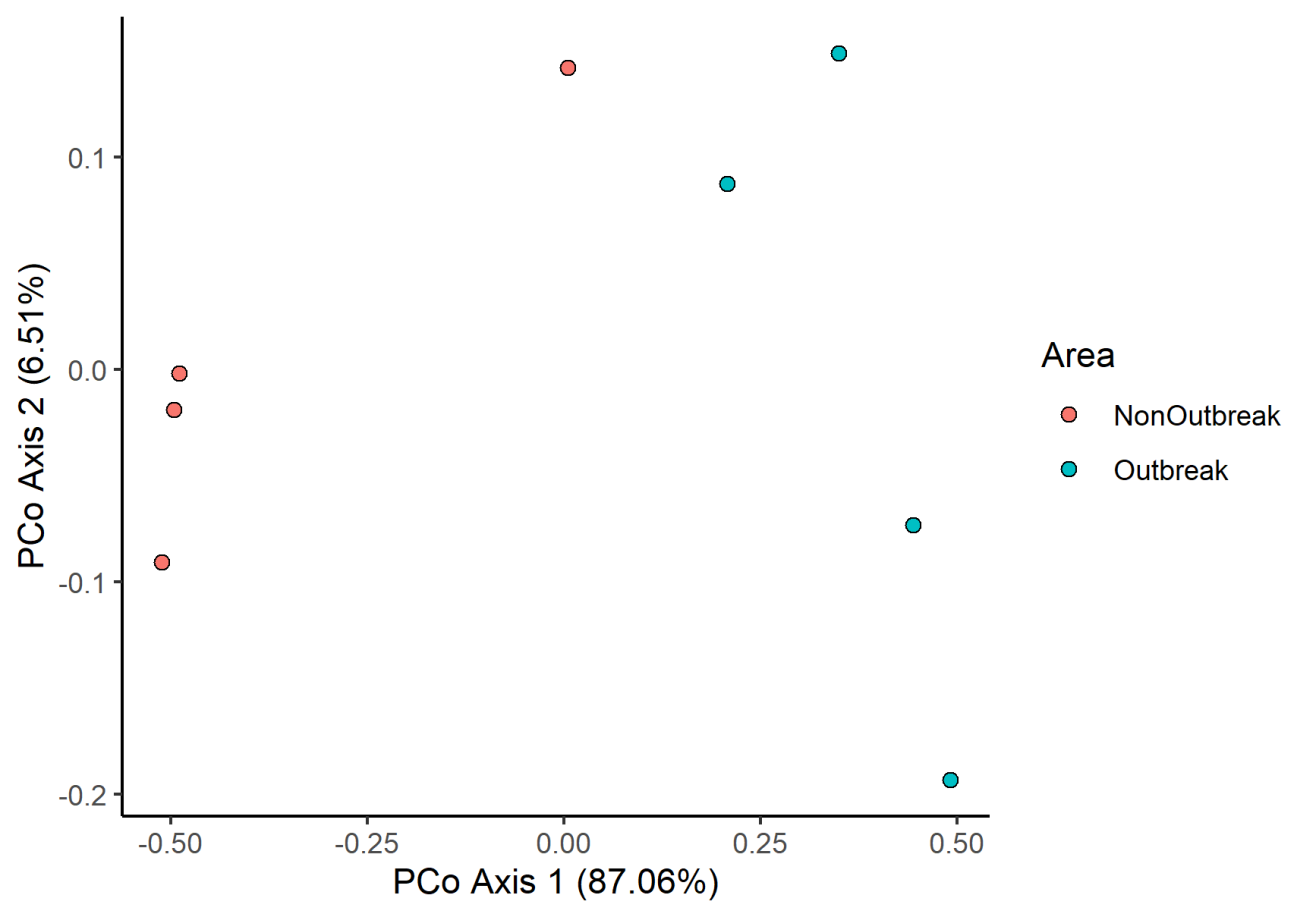
Principal Coordinate Analysis (PCOA) plot of bacterial communities of *M. plana* bagworm larvae in the comparison between areas.

From the PCoA (Fig. 6), we observed a clear separation between the samples from non-outbreak area and outbreak area. AMOVA test (Table 5) showed separation between the two areas was significantly different. This meant that the bacterial community structure was different from one another.

The HOMOVA test (Table 6) showed that there was no significant difference in the variation of bacterial community between the two areas. The non-outbreak area has a higher variation (0.063) compared to the outbreak area (0.027).

## DISCUSSION

At present, the microbiota of *M. plana* has yet to be uncovered. From the results, it was observed that the microbiota of *M. plana* was diverse but dominated by the phylum *Proteobacteria* and *Actinobacteria*, with a dominance of more than 97%. Nonetheless, the dominant phyla and other minor phyla such as *Actinobacteria, Bacterioidetes* and *Firmicutes* could be found in other lepidopteran such as silkworm *Bombyx mori* (Chen *et al*. 2018), oriental fruit moth *Grapholita molesta* (Yuan *et al*. 2021), cotton leafworm *Spodoptera littoralis* (Chen *et al*. 2016) and many other lepidopteran species compiled by (Voirol *et al*. 2018; Snyman *et al*. 2016). The presence of *Enterobacteriaceae, Microbacteriaceae*, *Burkholderiaceae*, *Pseudomonadaceae* and *Sphingobacteriaceae* were also observed in different lepidopteran studies (Jones *et al*. 2019; Robinson *et al*. 2010; Xia *et al*. 2013; Voirol *et al*. 2018). In terms of bacterial genus, *Pantoea, Curtobacterium, Pseudomonas* and *Massilia* genera found in the study were also found in other lepidopteran species (Robinson *et al*. 2010; Chen *et al*. 2018; Voirol *et al*. 2018; Jones *et al*. 2019).

Focusing on the most dominant genus found in this study, the *Pantoea*, could the dominance of this genus have any effect on the host *M. plana*? From literatures, a wide range of insect were observed to have relationship with different *Pantoae* species (Akhoundi *et al*. 2012; Aly *et al*. 2008; Asis & Adachi 2004; Azad *et al.* 2000; Walterson & Stavrinides 2015), with some relationship being mutualistic or commensalistic (Pinto-Tomás *et al*. 2009; Maccollom *et al*. 2009; Walterson & Stavrinides 2015). It was also reported that the *P. agglomerans* with another bacteria *Klebsiella pneumoniae* were able to mend the gut of irradiated Mediterranean fruit fly *Ceratitis capitata* and influencing the fitness of fruit fly fitness in a positive way (Niyazi *et al.* 2004; Maccollom *et al*. 2009). Furthermore, it was reported that *P. agglomerans* could fix atmospheric nitrogen (Vorwerk *et al.* 2007; Walterson & Stavrinides 2015). As previously mentioned, chewing insects that feed on leaves such as bagworm could not depend solely on their diet to get enough nitrogen (Hansen *et al.* 2020; Nardi *et al.* 2002; Voirol *et al*. 2018) and this nitrogen deficiency may be supplemented by *Pantoae which* can fix nitrogen and convert it into appropriate nitrogen-containing compounds. These examples of the benefits of *Pantoea* may have helped the bagworm to survive in the oil palm plantations.

Shifting the focus onto the next abundant genus, the *Curtobacterium*, it is said that the habitat of the Gram-positive, obligate aerobic chemoorganotrophs (Finn *et al*. 2014) mainly associated with plants and notably the phyllosphere (Behrendt *et al*. 2002; Chase *et al*. 2016; Komagata *et al.* 1965). In the genus, only the *C. flaccumfaciens* is linked to plant pathogenesis, while there are indications of other ecological roles performed by the other species of the genus such as endophytic symbionts (Bulgari *et al*. 2009), stimulate plant defence responses (Bulgari *et al*. 2011), reduce plant disease symptoms (Lacava *et al*. 2007), and even promote plant growth (Sturz *et al*. 1997). However, as the bacterium is mainly associated with plants, we believed that the bacterium does not contribute to the survivability of the bagworm and the bagworm merely obtain the bacterium from their diet without any benefits although further research is needed to prove this.

Nevertheless, there could also be a possibility that the larvae obtained these bacteria solely from their environment or diet but provided little or no benefit. Phalnikar *et al.* (2018) observed that the most common and abundant OTUs in butterflies were also common in different insect-associated microbiomes. This led them to hypothesise that the insect-bacterial co-occurrence may indicate evolved functional relationships, or it could merely act as ecological or dietary roles. The latter hypothesis might be due to absence or presence of very little resident bacteria found in caterpillar such as in a study done by Hammer *et al*. (2017) and is in agreement with Phalnikar *et al.* (2018) where they found a substantial overlap of bacterial communities from larval and dietary resources which indicated that bacterial communities in larval are mainly influenced by passive procurement of bacteria from dietary resources (Phalnikar *et al.* 2018). Furthermore, a study showed that insects that feed on foliar obtained their microbiomes from the soil (Hannula *et al*. 2019). The authors in the mentioned study stated that the microbiome of the caterpillar that fed on intact plant had a more distinct microbiome and the microbiome resembled the soil microbiomes. In another study, (Gomes *et al*. 2020) found that the caterpillar’s bacterial communities resembled the local soil microbiomes in which the host plant was growing. Nevertheless, it is important to note that the microbiome varies greatly across Lepidopteran species and even within species (Voirol *et al*. 2018). As the entire larvae were sampled, there was no trace as to where exactly these bacteria reside, although some studies had found that the bacterial communities from the whole insect can be similar to the bacterial communities sampled from the gut (Hammer *et al.* 2014; Voirol *et al*. 2018; Sabree *et al.* 2012; Sudakaran *et al*. 2012). Further studies to compare the microbiota of oil palm leaves and the bagworm microbiota is recommended in order to confirm if the bacteria found in this study is resident bacteria of bagworm.

In this study, we compared the bacterial community of bagworm of two developmental stages in outbreak area, and the bacterial community of bagworm from different areas. However, we did not observe any significant difference in the alpha and beta diversity for both comparisons. This phenomenon was also observed in some Lepidopteran species such as *Plodia interpunctella* and *Plutella xylostellai*, where their bacterial community did not change across developmental stages (Mereghetti *et al.* 2017; Ng *et al*. 2018; Voirol *et al*. 2018; Xia *et al*. 2018). The similarity in the bacterial community between developmental stage could also be attributed to the larvae having the same host plant (oil palm tree *Elaeis guineensis*), as different diet might influence bacterial communities in different ways such as promoting differential bacterial growth (Staudacher *et al*. 2016; Vorholt 2012; Yang *et al*. 2001). In regard to the comparison between areas where we observed no significant difference in alpha and beta diversity, a study found high consistency of the most dominant bacterial amplicon sequence variant (ASV) were detected in all the monophagous caterpillar *Tyria jacobaeae* across habitats regardless of their size (Gomes *et al*. 2020). In their study, Gomes *et al*. (2020) suggested that the fairly stable internal bacterial composition is possibly affected by the physiology of the caterpillar or an adaption to the exclusive diet of ragwort plants as well as phytochemicals. Following their observation, we could hypothesise that the same situation could have happen to the bacterial community of the bagworm from different areas.

This study provides a first insight to the bacterial community of the *M. plana* larvae and the information here may be of use for future management of the bagworm such as the use of biocontrol to control the outbreak. Nonetheless, it is still at the stage where more research is needed such as determining whether the bagworm microbiota was obtained from their diet or influenced by soil microbiota, which could be important if we wish to use biocontrol to target the resident microbiota of bagworm larvae. Furthermore, a metatranscriptomic analysis on the bacteria of the bagworm allows us to observe the gene expression profile of the complex microbial communities. This would allow us to see how the microbiome respond in the bagworm.

## Figures and Tables

**Table 1:** Alpha-diversity of the larvae of *M. plana* in comparison between instar stage.

Stage	Sample	Shannon	Sobs	Evenness
Early	OES4	1.361	194.736	0.258
	OES5	1.030	195.553	0.195
	OES6	2.204	322.000	0.382
	OES7	1.888	262.820	0.339
	Average	1.621	243.777	0.294
Late	OLS3	0.708	112.792	0.150
	OLS4	1.791	221.302	0.332
	OLS5	1.872	125.301	0.388
	OLS6	1.227	214.961	0.228
	Average	1.400	168.589	0.274
Wilcoxon test	*p*-value	0.486	0.343	0.886

**Table 2:** AMOVA test done on samples from early instar stage and late instar stage.

Early – End	Among	Within	Total
Sum of square (SS)	0.010	0.191	0.201
Degree of freedom (df)	1	6	7
Mean square (MS)	0.010	0.032	
F ratio (Fs)	0.325		
*p*-value: 0.554			

**Table 3:** HOMOVA test done on the samples from early instar stage and late instar stage.

HOMOVA	*p*-value	SSwithin/(Ni – 1) values
Early–Late	0.776	0.038 – 0.026

**Table 4:** Alpha-diversity of larvae of *M. plana* in the comparison between non-outbreak area and outbreak area.

Area	Sample	Shannon	Sobs	Evenness
Non-outbreak	NLS0	1.691	133.576	0.345
	NLS7	1.947	88.000	0.435
	NLS12	1.248	92.506	0.276
	NLS16	1.093	101.029	0.237
	Average	1.494	103.778	0.323
Outbreak	OLS3	0.678	50.042	0.173
	OLS4	1.773	104.775	0.381
	OLS5	1.893	87.202	0.424
	OLS6	1.156	89.667	0.257
	Average	1.375	82.922	0.309
Wilcoxon test	*p*-value	0.886	0.343	0.886

**Table 5:** AMOVA test done on samples from non-outbreak and outbreak area.

Non-outbreak–outbreak	Among	Within	Total
Sum of square (SS)	1.087	0.269	1.357
Degree of freedom (df)	1	6	7
Mean square (MS)	1.087	0.045	
F ratio (Fs)	24.209		
*p*-value: 0.034^*^			

**Table 6:** HOMOVA test done on the samples from non-outbreak and outbreak area.

HOMOVA	*p*-value	SSwithin/(Ni–1) values
Non-outbreak–outbreak	0.17	0.063–0.027

## APPENDICES

### Appendix A

Bacterial phyla of early instar stage and late instar stage of *M. plana* larvae from the outbreak area.

**Table:**

Phyla	Early instar stage (%)	Late instar stage (%)	Average relative abundance (%)	*p*-value adjusted
*Proteobacteria*	82.28	82.45	82.36	1.000
*Actinobacteria*	15.68	13.92	14.80	0.765
*Bacteroidetes*	1.26	1.70	1.48	0.765
*Firmicutes*	0.38	1.65	1.01	0.765
*Bacteria_unclassified*	0.19	0.12	0.16	0.765
*Planctomycetes*	0.18	0.14	0.16	1.000
*Patescibacteria*	0.03	0.02	0.02	0.807
*Verrucomicrobia*	0.00	0.00	0.00	1.000
*Acidobacteria*	0.00	0.00	0.00	0.765
*Deinococcus_Thermus*	0.00	0.00	0.00	0.765
*Chlamydiae*	0.00	0.00	0.00	0.765
*Nitrospirae*	0.00	0.00	0.00	0.765
*Tenericutes*	0.00	0.00	0.00	1.000

### Appendix B

Bacterial families of early instar stage and late instar stage of *M. plana* larvae from the outbreak area.

**Table:**

Family	Early instar stage (%)	Late instar stage (%)	Average relative abundance (%)	*p*-value adjusted
*Enterobscteriaceae*	75.29	75.46	75.37	0.965
*Microbacteriaceae*	14.77	12.50	13.63	0.965
*Burkholderiaceae*	4.40	2.49	3.44	0.741
*Pseudomonadaceae*	1.59	3.53	2.56	0.741
*Sphingobacteriaceae*	0.99	1.18	1.09	0.965
*Clostridiales_unclassified*	0.00	1.48	0.74	0.741
*Gammaproteobacterial_unclassified*	0.54	0.43	0.49	0.965
*Micrococcaceae*	0.07	0.61	0.34	1.000
*Weeksellaceae*	0.03	0.26	0.14	0.741
*Actinobacteria_unclassified*	0.11	0.13	0.12	0.912
*Cytophagales_unclassified*	0.16	0.08	0.12	0.912
*Rhodanobacteraceae*	0.03	0.18	0.11	0.965
*Corynebacteriaceae*	0.11	0.02	0.07	0.912
*Kineosporiaceae*	0.04	0.10	0.07	0.965
*Hymenobacteraceae*	0.04	0.08	0.06	0.848
*Micrococcales_unclassified*	0.08	0.04	0.08	0.965
*Intrasporangiaceae*	0.07	0.02	0.05	0.965
*Moraxellaceae*	0.03	0.06	0.05	0.912
*Propionobacteriaceae*	0.08	0.01	0.05	0.741
*Solirubrobacteraceae*	0.04	0.05	0.05	0.965
*Planococcaceae*	0.02	0.06	0.04	0.741
*Flavobacteriaceae*	0.00	0.06	0.03	0.741
*Staphylococcaceae*	0.04	0.02	0.03	1.000
*Spirosomaceae*	0.03	0.03	0.03	0.965
*Bacillaceae*	0.01	0.04	0.02	0.912
*P3OB-42*	0.03	0.02	0.02	0.741
*Nocardioidaceae*	0.00	0.04	0.02	0.741
*Parcubacteria_unclassified*	0.02	0.02	0.02	0.741
*Beijerinckiaceae*	0.02	0.01	0.02	0.965
*Legionellaceae*	0.03	0.00	0.01	0.741
*Vibrionaceae*	0.00	0.03	0.01	0.741
*Alphaproteobacteria_unclassified*	0.02	0.01	0.01	0.741
*Carnobacteriaceae*	0.03	0.00	0.01	0.741
*Dermabacteraceae*	0.00	0.02	0.01	0.741
*Acetobacteraceae*	0.02	0.00	0.01	0.741
*Rhizobiales_unclassified*	0.02	0.00	0.01	0.741
*Chitinophagaceae*	0.01	0.01	0.01	0.965
*0319-6G20*	0.01	0.01	0.01	0.741
*Streptococcaceae*	0.01	0.00	0.01	0.965
*Actinomycetaceae*	0.01	0.00	0.01	0.965
*Brevibacteriaceae*	0.01	0.00	0.01	0.741
*Xanthomonadaceae*	0.01	0.00	0.01	0.741
*Deltaproteobacteria_unclassified*	0.00	0.01	0.00	0.741
*Neisseriaceae*	0.00	0.01	0.00	0.965
*Betaproteobacteriales_unclassified*	0.01	0.00	0.00	0.741
*Cellulomonadaceae*	0.01	0.00	0.00	0.741
*Geodermatophilaceae*	0.01	0.00	0.00	0.741
*Frankiales_unclassified*	0.01	0.00	0.00	0.741
*Aeromonadaceae*	0.00	0.00	0.00	0.741
*NS11-12_marine_group*	0.00	0.00	0.00	0.741
*Rubritaleaceae*	0.00	0.00	0.00	0.741
*Diplorickettsiaceae*	0.00	0.00	0.00	0.741
*Planctomycetacia_unclassified*	0.00	0.00	0.00	0.741
*Acidobacteriaceae_(Subgroup_1)*	0.00	0.00	0.00	0.741
*Candidatus_Adlerbacteria_fa*	0.00	0.00	0.00	0.741
*Deinococcaceae*	0.00	0.00	0.00	0.741
*Erysipelotrichaceae*	0.00	0.00	0.00	0.741
*Gemmataceae*	0.00	0.00	0.00	0.741
*Pirellulaceae*	0.00	0.00	0.00	0.741
*Sporichthyaceae*	0.00	0.00	0.00	1.000
*Bacteroidaceae*	0.00	0.00	0.00	1.000
*Bacteroidia_unclassified*	0.00	0.00	0.00	0.741
*Enterococcaceae*	0.00	0.00	0.00	1.000
*Nitrosomonadaceae*	0.00	0.00	0.00	0.741
*Nitrospiraceae*	0.00	0.00	0.00	0.741
*Parachlamydiaceae*	0.00	0.00	0.00	0.741
*Parcubacteria_fa*	0.00	0.00	0.00	0.741
*Saccharimonadales_unclassified*	0.00	0.00	0.00	0.741
*Streptomycetaceae*	0.00	0.00	0.00	0.741
*Veillonellaceae*	0.00	0.00	0.00	0.741
*Bacteriovoracaceae*	0.00	0.00	0.00	0.741
*Candidatus_Peribacteria_fa*	0.00	0.00	0.00	0.741
*Crocinitomicaceae*	0.00	0.00	0.00	0.741
*Micromonosporaceae*	0.00	0.00	0.00	0.741
*Mycoplasmataceae*	0.00	0.00	0.00	0.741
*Rhodocyclaceae*	0.00	0.00	0.00	0.741
*Thermoleophilia_unclassified*	0.00	0.00	0.00	0.741

### Appendix C

Bacterial genera of early instar stage and late instar stage of *M. plana* larvae from the outbreak area.

**Table:**

Genus	Early instar stage (%)	Late instar stage (%)	Average relative abundance (%)	p-value adjusted
*Pantoea*	56.76	64.38	60.57	0.999
*Enterobacteriaceae_*	18.45	11.00	14.72	0.657
*Curtobacterium*	13.59	12.12	12.86	1.000
*Pseudomonas*	1.59	3.53	2.56	0.657
*Massilia*	3.08	1.72	2.40	0.657
*Burkholderia-Caballeronia-Paraburkholderia*	1.25	0.60	0.92	0.657
*Sphingobacteriaceae_*	0.71	1.04	0.88	0.837
*Microbacteriaceae_*	1.15	0.34	0.74	0.657
*Clostridiales_Clostridiales_*	0.00	1.48	0.74	0.657
*Gammaproteobacteria_Gammaproteobacteria_Gammaproteobacteria_*	0.54	0.43	0.49	0.99
*Arthrobacter*	0.00	0.58	0.29	1.000
*Mucilaginibacter*	0.27	0.14	0.21	1.000
*Proteobacteria_Proteobacteria_Proteobacteria_Proteobacteria_*	0.20	0.19	0.20	1.000
*Pseudonocardia*	0.04	0.28	0.16	0.657
*Bacteria_Bacteria_Bacteria_Bacteria_Bacteria_*	0.19	0.12	0.16	0.657
*Lactobacillus*	0.27	0.04	0.16	0.837
*Mycobacterium*	0.20	0.08	0.14	0.657
*Chryseobacterium*	0.02	0.26	0.14	0.657
*Cytophagales_Cytophagales_*	0.16	0.08	0.12	0.837
*Actinobacteria_Actinobacteria_Actinobacteria_*	0.10	0.13	0.12	0.837
*Rhodanobacter*	0.03	0.18	0.11	0.999
*Singulisphaera*	0.10	0.09	0.09	0.837
*Burkholderiaceae_*	0.04	0.10	0.07	0.837
*Kineococcus*	0.04	0.10	0.07	0.999
*Corynebacterium_1*	0.11	0.02	0.06	0.657
*Escherichia-Shigella*	0.04	0.08	0.06	0.963
*Hymenobacter*	0.04	0.08	0.06	0.765
*Isosphaeraceae_*	0.07	0.05	0.06	1.000
*Micrococcales_Micrococcales_*	0.08	0.04	0.06	0.999
*Cutibacterium*	0.08	0.01	0.05	0.657
*Solirubrobacter*	0.04	0.05	0.05	0.999
*Tetrasphaera*	0.05	0.02	0.04	0.999
*Acinetobacter*	0.03	0.05	0.04	0.999
*Flavobacterium*	0.00	0.06	0.03	0.657
*Aquabacterium*	0.02	0.05	0.03	0.999
*Staphylococcus*	0.04	0.02	0.03	1.000
*Kocuria*	0.03	0.02	0.03	0.832
*Bacillus*	0.01	0.04	0.02	0.837
*P3OB-42_ge*	0.03	0.02	0.02	0.657
*Spirosomaceae_*	0.02	0.02	0.02	0.999
*Microbacterium*	0.02	0.03	0.02	1.000
*Parcubacteria_Parcubacteria_Parcubacteria_*	0.02	0.02	0.02	0.657
*Planococcus*	0.00	0.04	0.02	0.657
*Nocardioides*	0.00	0.04	0.02	0.657
*Kosakonia*	0.02	0.01	0.01	0.657
*Legionella*	0.03	0.00	0.01	0.657
*Vibrio*	0.00	0.03	0.01	0.657
*Alloiococcus*	0.03	0.00	0.01	0.657
*Alphaproteobacteria_Alphaproteobacteria_Alphaproteobacteria_*	0.02	0.01	0.01	0.657
*Sporosarcina*	0.01	0.02	0.01	1.000
*Fodinicola*	0.02	0.01	0.01	0.999
*Sphingomonadaceae_*	0.02	0.00	0.01	0.657
*Brachybacterium*	0.00	0.02	0.01	0.657
*Rothia*	0.02	0.00	0.01	0.657
*Micrococcaceae_*	0.01	0.00	0.01	0.698
*Rhizobiales_Rhizobiales_*	0.02	0.00	0.01	0.657
*Beijerinckiaceae_*	0.01	0.01	0.01	0.657
*Acetobacteraceae_*	0.01	0.00	0.01	0.657
*Enhydrobacter*	0.01	0.01	0.01	1.000
*Methylobacterium*	0.01	0.01	0.01	1.000
*Intrasporangiaceae_*	0.01	0.00	0.01	0.657
*Spirosoma*	0.00	0.01	0.01	0.832
*0319-6G20_ge*	0.01	0.01	0.01	0.657
*Acidovorax*	0.00	0.01	0.01	0.657
*Serratia*	0.01	0.00	0.01	0.657
*Streptococcus*	0.01	0.00	0.01	0.999
*Brevibacterium*	0.01	0.00	0.01	0.657
*Leucobacter*	0.00	0.01	0.01	0.657
*Corynebacterium*	0.01	0.00	0.00	0.657
*Deltaproteobacteria_Deltaproteobacteria_Deltaproteobacteria_*	0.00	0.01	0.00	0.657
*Lysinibacillus*	0.01	0.00	0.00	0.657
*Delftia*	0.01	0.00	0.00	0.657
*Betaproteobacteriales_Betaproteoacteriales_*	0.01	0.00	0.00	0.657
*Actinomyces*	0.01	0.00	0.00	0.657
*Cellulomonadaceae_*	0.01	0.00	0.00	0.657
*Chitinophaga*	0.01	0.00	0.00	0.657
*Geodermatophilus*	0.01	0.00	0.00	0.657
*Pedobacter*	0.01	0.00	0.00	0.657
*Neisseriaceae_*	0.00	0.01	0.00	1.000
*Yonghaparkia*	0.01	0.00	0.00	0.657
*Xanthomonadaceae_*	0.01	0.00	0.00	0.657
*Frankiales_Frankiales_*	0.01	0.00	0.00	0.657
*Chitinophagaceae_*	0.00	0.01	0.00	0.657
*Luteolibacter*	0.00	0.00	0.00	0.657
*Comamonas*	0.00	0.00	0.00	0.657
*Corynebacteriaceae_*	0.00	0.00	0.00	0.657
*NS11-12_marine_group_ge*	0.00	0.00	0.00	0.657
*Rhodoluna*	0.00	0.00	0.00	0.657
*Trueperella*	0.00	0.00	0.00	0.657
*Aeromonas*	0.00	0.00	0.00	0.657
*Ellin6055*	0.00	0.00	0.00	1.000
*Planctomycetacia_Planctomycetaca_Planctomycetacia_*	0.00	0.00	0.00	0.657
*Stenotrophomonas*	0.00	0.00	0.00	0.657
*Taibaiella*	0.00	0.00	0.00	0.657
*Candidatus_Adlerbacteria_ge*	0.00	0.00	0.00	0.657
*Deinococcus*	0.00	0.00	0.00	0.657
*Pirellula*	0.00	0.00	0.00	0.657
*Turicibacter*	0.00	0.00	0.00	0.657
*uncultured*	0.00	0.00	0.00	0.657
*hgcI_clade*	0.00	0.00	0.00	1.000
*Lelliottia*	0.00	0.00	0.00	0.657
*Nocardioidaceae_*	0.00	0.00	0.00	1.000
*Bacteroides*	0.00	0.00	0.00	1.000
*Cloacibacterium*	0.00	0.00	0.00	0.657
*Enterococcus*	0.00	0.00	0.00	1.000
*Ralstonia*	0.00	0.00	0.00	0.657
*Acetobacter*	0.00	0.00	0.00	0.657
*Actinobacteria_Actinobacteria_Actiobacteria_Actinobacteria_*	0.00	0.00	0.00	0.657
*Arenimonas*	0.00	0.00	0.00	0.657
*Bacteroidia_Bacteroidia_Bacteroidia_*	0.00	0.00	0.00	0.657
*Bryocella*	0.00	0.00	0.00	0.657
*Candidatus_Protochlamydia*	0.00	0.00	0.00	0.657
*Diaphorobacter*	0.00	0.00	0.00	0.657
*Diplorickettsiaceae_*	0.00	0.00	0.00	0.657
*Hymenobacteraceae_*	0.00	0.00	0.00	0.657
*Micrococcus*	0.00	0.00	0.00	0.657
*Neisseria*	0.00	0.00	0.00	0.657
*Nitrosomonas*	0.00	0.00	0.00	0.657
*Nitrospira*	0.00	0.00	0.00	0.657
*Parcubacteria_ge*	0.00	0.00	0.00	0.657
*Pseudacidovorax*	0.00	0.00	0.00	0.657
*Pseudonocardiaceae_*	0.00	0.00	0.00	0.657
*Rickettsiella*	0.00	0.00	0.00	0.657
*Saccharimonadales_Saccharimonadales_*	0.00	0.00	0.00	0.657
*Streptomycetaceae_*	0.00	0.00	0.00	0.657
*Veillonellaceae_*	0.00	0.00	0.00	0.657
*Achromobacter*	0.00	0.00	0.00	0.657
*Acidobacteriaceae_(Subgroup_1)_*	0.00	0.00	0.00	0.657
*Alcaligenes*	0.00	0.00	0.00	0.657
*Alicycliphilus*	0.00	0.00	0.00	0.657
*Candidatus_Peribacteria_ge*	0.00	0.00	0.00	0.657
*Capnocytophaga*	0.00	0.00	0.00	0.657
*Erwinia*	0.00	0.00	0.00	0.657
*Fluviicola*	0.00	0.00	0.00	0.657
*Legionellaceae_*	0.00	0.00	0.00	0.657
*Limnohabitans*	0.00	0.00	0.00	0.657
*Methyloversatilis*	0.00	0.00	0.00	0.657
*Micromonospora*	0.00	0.00	0.00	0.657
*Mycoplasma*	0.00	0.00	0.00	0.657
*Peredibacter*	0.00	0.00	0.00	0.657
*Thermoleophilia_Thermoleophilia_Thermoleophilia_*	0.00	0.00	0.00	0.657

### Appendix D

Bacterial phyla of the late instar stage of *M. plana* larvae from non-outbreak area and outbreak area.

**Table:**

Phyla	Non-outbreak area (%)	Outbreak area (%)	Average relative abundance (%)	*p*-value-adjusted
*Proteobacteria*	20.57	82.02	51.30	0.197
*Actinobacteria*	76.29	14.16	45.22	0.197
*Bacteroidetes*	2.19	1.76	1.98	0.720
*Firmicutes*	0.10	1.71	0.91	0.302
*Bacteria_unclassified*	0.60	0.14	0.37	0.302
*Planctomycetes*	0.03	0.15	0.09	0.720
*Patescibacteria*	0.05	0.03	0.04	1.000
*Verrucomicrobia*	0.07	0.00	0.03	0.302
*Chlamydiae*	0.05	0.00	0.02	0.302
*Cyanobacteria*	0.03	0.00	0.02	0.302
*Acidobacteria*	0.02	0.00	0.01	0.302
*Deinococcus-Thermus*	0.00	0.01	0.01	0.589
*Nitrospirae*	0.00	0.01	0.00	0.589

### Appendix E

Bacterial families of the late instar stage of *M. plana* larvae from non-outbreak area and outbreak area.

**Table:**

Family	Non-outbreak area (%)	Outbreak area (%)	Average relative abundance (%)	*p*-value adjusted
*Enterobacteriaceae*	11.67	75.41	43.54	0.550
*Microbacteriaceae*	70.87	12.47	41.67	0.550
*Pseudomonadaceae*	5.03	3.34	4.18	0.963
*Burkholderiaceae*	2.93	2.56	2.74	0.599
*Sphingobacteriaceae*	1.57	1.24	1.40	0.600
*Kineosporiaceae*	2.33	0.15	1.24	0.599
*Clostridiales_unclassified*	0.00	1.55	0.77	0.599
*Pseudonocardiaceae*	0.89	0.36	0.62	0.963
*Actinobacteria_unclassified*	0.95	0.16	0.55	0.599
*Micrococcaceae*	0.16	0.60	0.38	0.784
*Bacteria_unclassified*	0.60	0.14	0.37	0.599
*Weeksellaceae*	0.23	0.26	0.25	0.599
*Micrococcales_unclassified*	0.34	0.07	0.20	0.599
*Cytophagales_unclassified*	0.26	0.11	0.19	0.963
*Gammaproteobacteria_unclassified*	0.04	0.24	0.14	0.599
*Nocardioidaceae*	0.22	0.06	0.14	0.599
*Proteobacteria_unclassified*	0.10	0.15	0.13	0.600
*Beijerinckiaceae*	0.21	0.02	0.11	0.599
*Intrasporangiaceae*	0.16	0.03	0.09	0.784
*Isosphaeraceae*	0.01	0.15	0.08	0.599
*Rhodanobacteraceae*	0.00	0.16	0.08	0.599
*Geodermatophilaceae*	0.14	0.00	0.07	0.599
*Sphingomonadaceae*	0.12	0.01	0.07	0.550
*Bacillaceae*	0.06	0.06	0.06	0.963
*Moraxellaceae*	0.05	0.06	0.05	0.784
*P3OB-42*	0.10	0.00	0.05	0.550
*Xanthomonadaceae*	0.10	0.00	0.05	0.599
*Mycobacteriaceae*	0.00	0.09	0.05	0.599
*Corynebacteriaceae*	0.06	0.03	0.04	1.000
*Flavobacteriaceae*	0.01	0.07	0.04	0.599
*Parcubacteria_unclassified*	0.05	0.03	0.04	1.000
*Propionibacteriaceae*	0.05	0.03	0.04	0.963
*Hymenobacteraceae*	0.03	0.05	0.04	0.777
*Spirosomaceae*	0.04	0.03	0.03	0.963
*Alphaproteobacteria_unclassified*	0.06	0.00	0.03	0.550
*Lactobacillaceae*	0.01	0.05	0.03	0.599
*Solirubrobacteraceae*	0.00	0.06	0.03	0.599
*Cryptosporangiaceae*	0.04	0.01	0.03	1.000
*Streptomycetaceae*	0.05	0.00	0.03	0.599
*Chitinophagaceae*	0.05	0.00	0.02	0.599
*Planococcaceae*	0.00	0.05	0.02	0.599
*Rhizobiales_unclassified*	0.04	0.01	0.02	0.599
*Archangiaceae*	0.03	0.00	0.02	0.599
*Oxyphotobacteria_unclassified*	0.03	0.00	0.02	0.599
*Parachlamydiaceae*	0.03	0.00	0.02	0.599
*Rubritaleaceae*	0.03	0.00	0.01	0.599
*0319-6G20*	0.02	0.01	0.01	1.000
*Aeromonadaceae*	0.00	0.02	0.01	0.599
*Beggiatoaceae*	0.02	0.00	0.01	0.599
*Caulobacteraceae*	0.02	0.00	0.01	0.599
*Gemmataceae*	0.02	0.00	0.01	0.599
*Leuconostocaceae*	0.02	0.00	0.01	0.599
*Neisseriaceae*	0.01	0.01	0.01	1.000
*Verrucomicrobiaceae*	0.02	0.00	0.01	0.599
*Acidobacteriaceae_(Subgroup_1)*	0.02	0.00	0.01	0.599
*Betaproteobacteriales_unclassified*	0.02	0.00	0.01	0.599
*Brevibacteriaceae*	0.02	0.00	0.01	0.599
*Chthoniobacteraceae*	0.02	0.00	0.01	0.599
*Dermabacteraceae*	0.00	0.02	0.01	0.599
*Micromonosporaceae*	0.00	0.02	0.01	0.599
*Nakamurellaceae*	0.02	0.00	0.01	0.599
*Staphylococcaceae*	0.01	0.01	0.01	1.000
*Vibrionaceae*	0.00	0.02	0.01	0.599
*Acetobacteraceae*	0.01	0.00	0.01	0.599
*Chlamydiales_unclassified*	0.01	0.00	0.01	0.599
*Deinococcaceae*	0.00	0.01	0.01	0.599
*Legionellaceae*	0.00	0.01	0.01	0.599
*Actinomycetaceae*	0.00	0.01	0.00	0.599
*Amoebophilaceae*	0.01	0.00	0.00	0.599
*Bacillales_unclassified*	0.01	0.00	0.00	0.599
*Deltaproteobacteria_unclassified*	0.00	0.01	0.00	0.599
*Nitrospiraceae*	0.00	0.01	0.00	0.599
*Rhodobacteraceae*	0.01	0.00	0.00	0.599
*Veillonellaceae*	0.00	0.01	0.00	0.599

### Appendix F

Bacterial genera of the late instar stage of *M. plana* larvae from non-outbreak area and outbreak area.

**Table:**

Genus	Non-outbreak area (%)	Outbreak area (%)	Average relative abundance (%)	*p*-value adjusted
*Curtobacterium*	68.46	12.03	40.24	0.451
*Pantoea*	9.88	64.70	37.29	0.451
*Enterobacteriaceae_*	1.64	10.64	6.14	0.589
*Pseudomonas*	5.03	3.34	4.18	0.979
*Massilia*	1.15	1.67	1.41	0.795
*Sphingobacteriaceae_*	1.40	1.08	1.24	0.597
*Kineococcus*	2.33	0.15	1.24	0.589
*Burkholderia_Caballeronia_Paraburkholderia*	1.65	0.68	1.17	0.589
*Microbacteriaceae_*	1.78	0.23	1.01	0.451
*Clostridiales_Clostridiales_*	0.00	1.55	0.77	0.589
*Actinobacteria_Actinobacteria_Actinobacteria_*	0.94	0.16	0.55	0.589
*Pseudonocardia*	0.56	0.36	0.46	0.795
*Bacteria_Bacteria_Bacteria_Bacteria_Bacteria_*	0.60	0.14	0.37	0.589
*Arthrobacter*	0.00	0.60	0.30	0.589
*Yonghaparkia*	0.34	0.18	0.26	0.589
*Chryseobacterium*	0.23	0.26	0.25	0.589
*Micrococcales_Micrococcales_*	0.34	0.07	0.20	0.589
*Cytophagales_Cytophagales_*	0.26	0.11	0.19	0.979
*Pseudonocardiaceae_*	0.33	0.00	0.16	0.451
*Microbacterium*	0.30	0.02	0.16	0.979
*Gammaproteobacteria_Gammaproteobacteria_Gammaproteobacteria*	0.04	0.24	0.14	0.589
*Nocardioides*	0.21	0.05	0.13	0.589
*Proteobacteria_Proteobacteria_Proteobacteria_Proteobacteria_*	0.10	0.15	0.13	0.597
*Escherichia_Shigella*	0.15	0.06	0.11	0.589
*Intrasporangiaceae_*	0.16	0.03	0.09	0.789
*Burkholderiaceae_*	0.03	0.15	0.09	0.589
*Beijerinckiaceae_*	0.18	0.00	0.09	0.589
*Mucilaginibacter*	0.00	0.16	0.08	0.451
*Rhodanobacter*	0.00	0.16	0.08	0.589
*Sphingobacterium*	0.15	0.00	0.07	0.589
*Geodermatophilus*	0.14	0.00	0.07	0.589
*Sphingomonadaceae_*	0.12	0.01	0.07	0.483
*Singulisphaera*	0.01	0.11	0.06	1.000
*Kocuria*	0.11	0.01	0.06	0.589
*Bacillus*	0.05	0.06	0.05	1.000
*Aquabacterium*	0.06	0.04	0.05	0.675
*Stenotrophomonas*	0.10	0.00	0.05	0.589
*Acinetobacter*	0.05	0.05	0.05	0.895
*P3OB-42_ge*	0.10	0.00	0.05	0.451
*Mycobacterium*	0.00	0.09	0.05	0.589
*Cutibacterium*	0.05	0.03	0.04	0.979
*Flavobacterium*	0.01	0.07	0.04	0.589
*Parcubacteria_Parcubacteria_Parcubacteria_*	0.05	0.03	0.04	1.000
*Corynebacterium_1*	0.06	0.02	0.04	0.894
*Hymenobacter*	0.03	0.05	0.04	0.767
*Spirosomaceae_*	0.04	0.02	0.03	1.000
*Alphaproteobacteria_Alphaproteobacteria_Alphaproteobacteria_*	0.06	0.00	0.03	0.451
*Lactobacillus*	0.01	0.05	0.03	0.589
*Solirubrobacter*	0.00	0.06	0.03	0.589
*Fodinicola*	0.04	0.01	0.03	1.000
*Streptomyces*	0.05	0.00	0.03	0.589
*Micrococcus*	0.05	0.00	0.02	0.589
*uncultured*	0.05	0.00	0.02	0.589
*Methylobacterium*	0.03	0.02	0.02	0.979
*Rhizobiales_Rhizobiales_*	0.04	0.01	0.02	0.589
*Taibaiella*	0.05	0.00	0.02	0.589
*Isosphaeraceae_*	0.00	0.05	0.02	0.589
*Cystobacter*	0.03	0.00	0.02	0.589
*Planococcus*	0.00	0.03	0.02	0.589
*Candidatus_Protochlamydia*	0.03	0.00	0.02	0.589
*Oxyphotobacteria_Oxyphotobacteria_Oxyphotobacteria_*	0.03	0.00	0.02	0.589
*Luteolibacter*	0.03	0.00	0.01	0.589
*0319-6G20_ge*	0.02	0.01	0.01	1.000
*Acidovorax*	0.00	0.02	0.01	0.589
*Aeromonas*	0.00	0.02	0.01	0.589
*Candidatus_Maribeggiatoa*	0.02	0.00	0.01	0.589
*Gemmataceae_*	0.02	0.00	0.01	0.589
*Neisseriaceae_*	0.01	0.01	0.01	1.000
*Comamonas*	0.01	0.01	0.01	1.000
*Staphylococcus*	0.01	0.01	0.01	1.000
*Betaproteobacteriales_Betaproteobacteriales_*	0.02	0.00	0.01	0.589
*Brachybacterium*	0.00	0.02	0.01	0.589
*Brevibacterium*	0.02	0.00	0.01	0.589
*Candidatus_Udaeobacter*	0.02	0.00	0.01	0.589
*Micromonospora*	0.00	0.02	0.01	0.589
*Nakamurella*	0.02	0.00	0.01	0.589
*Pedobacter*	0.02	0.00	0.01	0.589
*Vibrio*	0.00	0.02	0.01	0.589
*Nocardioidaceae_*	0.01	0.01	0.01	1.000
*Acetobacteraceae_*	0.01	0.00	0.01	0.589
*Acidobacteriaceae_(Subgroup_1)_*	0.01	0.00	0.01	0.589
*Actinobacteria_Actinobacteria_Acti nobacteria_Actinobacteria_*	0.01	0.00	0.01	0.589
*Chlamydiales_Chlamydiales_*	0.01	0.00	0.01	0.589
*Corynebacteriaceae_*	0.00	0.01	0.01	0.589
*Deinococcus*	0.00	0.01	0.01	0.589
*Kosakonia*	0.00	0.01	0.01	0.589
*Legionella*	0.00	0.01	0.01%	0.589
*Leucobacter*	0.00	0.01	0.01%	0.589
*Leuconostoc*	0.01	0.00	0.01%	0.589
*Ralstonia*	0.01	0.00	0.01%	0.589
*Sporosarcina*	0.00	0.01	0.01%	0.589
*Weissella*	0.01	0.00	0.01%	0.589
*Bacillaceae_*	0.01	0.00	0.00%	0.589
*Bacillales_Bacillales_*	0.01	0.00	0.00%	0.589
*Bryocella*	0.01	0.00	0.00%	0.589
*Candidatus_Cardinium*	0.01	0.00	0.00%	0.589
*Deltaproteobacteria_Deltaproteobacteria_Deltaproteobacteria_*	0.00	0.01	0.00%	0.589
*Enhydrobacter*	0.00	0.01	0.00%	0.589
*Erwinia*	0.00	0.01	0.00%	0.589
*Nitrospira*	0.00	0.01	0.00%	0.589
*Rhodobacteraceae_*	0.01	0.00	0.00%	0.589
*Spirosoma*	0.00	0.01	0.00%	0.589
*Trueperella*	0.00	0.01	0.00%	0.589
*Veillonellaceae_*	0.00	0.01	0.00%	0.589
